# Behavior and abundance of *Anopheles darlingi* in communities living in the Colombian Amazon riverside

**DOI:** 10.1371/journal.pone.0213335

**Published:** 2019-03-07

**Authors:** César Camilo Prado, Luis Antonio Alvarado-Cabrera, Paola Andrea Camargo-Ayala, Diego Garzón-Ospina, Milena Camargo, Sara Cecilia Soto-De León, Juan Ricardo Cubides, Carmen Teresa Celis-Giraldo, Manuel Elkin Patarroyo, Manuel Alfonso Patarroyo

**Affiliations:** 1 Molecular Biology and Immunology Department, Fundación Instituto de Inmunología de Colombia (FIDIC), Bogotá, Colombia; 2 MSc Programme in Epidemiology, School of Medicine and Health Sciences, Universidad del Rosario, Bogotaá, Colombia; 3 PhD Programme in Biomedical and Biological Sciences, School of Medicine and Health Sciences, Universidad del Rosario, Bogotá, Colombia; 4 Universidad de Ciencias Aplicadas y Ambientales (UDCA), Bogotá, Colombia; 5 School of Medicine, Universidad Nacional de Colombia, Bogotá, Colombia; 6 Basic Sciences Department, School of Medicine and Health Sciences, Universidad del Rosario, Bogotá, Colombia; Johns Hopkins University Bloomberg School of Public Health, UNITED STATES

## Abstract

In the past few years, relative frequencies of malaria parasite species in communities living in the Colombian Amazon riverside have changed, being *Plasmodium vivax* (61.4%) and *Plasmodium malariae* (43.8%) the most frequent. Given this epidemiological scenario, it is important to determine the species of anophelines involved in these parasites’ transmission. This study was carried out in June 2016 in two indigenous communities living close to the tributaries of the Amazon River using protected human bait. The results of this study showed a total abundance of 1,085 mosquitos, of which 99.2% corresponded to *Anopheles darlingi*. Additionally, only two anopheline species were found, showing low diversity in the study areas. Molecular confirmation of some individuals was then followed by evolutionary analysis by using the *COI* gene. Nested PCR was used for identifying the three *Plasmodium* species circulating in the study areas. Of the two species collected in this study, 21.0% of the *An*. *darlingi* mosquitoes were infected with *P*. *malariae*, 21.9% with *P*. *vivax* and 10.3% with *Plasmodium falciparum*. It exhibited exophilic and exophagic behavior in both study areas, having marked differences regarding its abundance in each community (Tipisca first sampling 49.4%, Tipisca second sampling 39.6% and Doce de Octubre 10.9%). Interestingly, *An*. *mattogrossensis* infected by *P*. *vivax* was found for the first time in Colombia (in 50% of the four females collected). Analysis of *An*. *darlingi COI* gene diversity indicated a single population maintaining a high gene flow between the study areas. The *An*. *darlingi* behavior pattern found in both communities represents a risk factor for the region’s inhabitants living/working near these sites. This highlights the need for vector control efforts such as the use of personal repellents and insecticides for use on cattle, which must be made available in order to reduce this Anopheline’s abundance.

## Introduction

Malaria is the parasitic disease with the greatest worldwide impact. Reports of cases increased by 2 million in 2017 with respect to the previous year, for a total of 219.000 million cases worldwide. In the Americas, case number increases have been reported in the past three years with respect to 2015, largely due to an increase in malaria transmission in Venezuela, Brazil and Nicaragua [1]. Up to the year 2010, *Plasmodium vivax* had been reported as the most prevalent malaria parasite in Colombia [2]. However, there has been an increase in *P*. *falciparum* and *P*. *malariae* prevalence since 2014 in some parts of the country [3]. Four regions have been recognized as being the main focal points of malarial transmission in Colombia: the region between the lower Cauca, Sinú and Urabá river basins in the northeast, the Pacific coast in the west, part of the Orinoquía region in the east of the country and the Amazon region in the south [4]. There has been an increase in cases in the latter region in the past four years [5–7]. Recent studies highlighted high *P*. *malariae* circulation along the banks of the Amazon and Loretoyacu rivers, in addition to *P*. *vivax* and *P*. *falciparum* [8,9].

*Plasmodium* spp. species parasitizing humans are transmitted by the bite of female mosquitoes from the *Anopheles* spp. genus [10]. Around 440 species of *Anopheles* mosquitoes have been described worldwide, 70 of them being potential malarial vectors [11,12]. In Colombia, about 47 Anopheline species have been recorded, 9 of them considered primary and/or secondary vectors of the disease [13]. Historically, *Anopheles albimanus*, *An*. *darlingi* and *An*. *nuneztovary* have been recognized as primary vectors for the whole of Colombia, while *An*. *darlingi* is the primary vector in the Amazon region. However, studies carried out in the same area have found *An*. *rangeli*, *An*. *oswaldoi* and *An*. *benarrochi* specimens naturally infected with *P*. *vivax*, which could be potential vectors for malaria transmission [14–16]. Nevertheless, studies aimed at understanding malaria transmission dynamics emphasizing the vectors involved in its dispersion are still lacking, especially in mosquito species which might be *P*. *malariae* vectors [14,17]. These entomological studies would allow us to understand the relationship between climate conditions, the parasite and the vector, leading to a realistic and significant assessment of the impact of climate change and other social factor on malaria transmission in the Colombian Amazon [18].

This study was thus aimed at analyzing the pertinent entomological scenario, the taxonomic and molecular identification of the *Anopheles* species and determining the infection frequency of three species causing human malaria (*P*. *vivax*, *P*. *malariae* and *P*. *falciparum*) in the weeks prior to the periods of high transmission (historically reported to start in the year’s second period) in two communities living in Colombia’s Amazonas department [19].

## Materials and methods

### Study population

**Fig 1:**
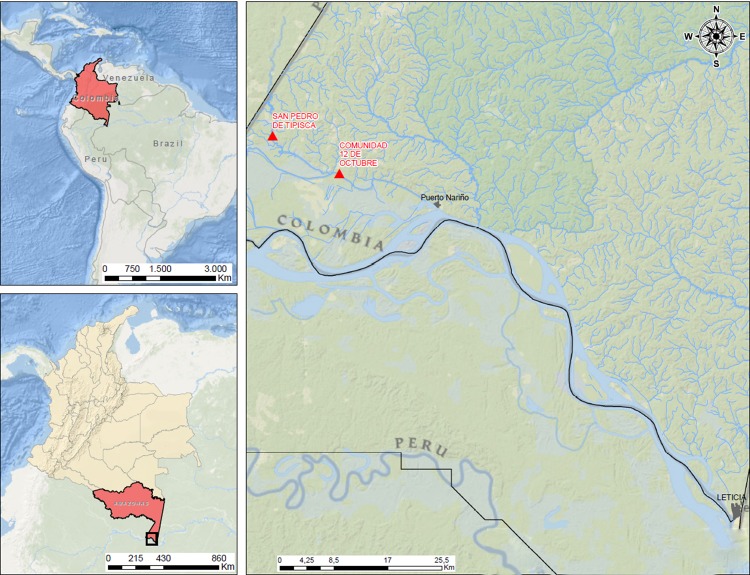
Map. Geographical location of both communities where mosquitoes were collected (the map has been modified from an Instituto Geográfico Agustín Codazzi (IGAC) template [20]. The images are open access, accessible and modifiable, according to IGAC policy.

This study was carried out during June 2016 in two epidemiologically relevant indigenous communities living in Colombia’s Amazonas department in terms of capturing mosquitoes, vector-borne diseases, proximity to the tributaries of the Amazon River, accessibility regarding sampling and vector-capture personnel’s safety. Samples (n = 3) were obtained over three consecutive days. Sampling was carried out in the Tipisca community on two occasions (Tp1 and Tp2) (3°41'49.96''S; 70°35:06.42''W), and once in the Doce de Octubre (DO) community (3°44'14.04'' S; 70°30'08.45'' W). A dwelling was chosen in each community according to local health workers’ recommendations as their knowledge of local dynamics indicated the area having the greatest abundance of mosquitoes (Fig 1).

### Ethical considerations

This study was framed within a project known as, “Malaria prevention and control strategies in the Amazon region in response to a recent outbreak of the disease” (BPIN-266 project, special cooperation agreement N° 0020 between the Amazonas governorate–FIDIC) which was approved and supervised by the Universidad del Rosario’s School of Medicine and Health Sciences’ Ethical Research Committee (Colombia: resolution CEI-ABN 026–000161). Resolution 530 (27^th^ May 2014) led to the administrative act allowing the mosquito collection for scientific research purposes.

### Mosquito capture, storage and transport

Specimens were captured on three consecutive days. The methodological design envisaged sampling periods for each community covering 6 hours continuously (06:00 pm to 11:00 pm), taking 50 minutes for capturing specimens and 10 minutes for counting them.

In each sampling area, three ecotopes were used to capture mosquitos, separated by 10–20 meters from each other. A collector was placed at each of the following sampling sites to guarantee the simultaneous collection of individuals in each of the ecotopes: intradomiciliary (sampling within the study dwelling), peridomiciliary (outside the house but within a distance of 10 m) and extradomiciliary (the area located 20 meters from the intradomiciliary area). Captures using the protected human bait technique were made in the ecotopes as this is considered the most used methodology for collecting hematophagous insects [21,22].

Each collector carried an informed consent form which had been reviewed and approved by the Ethics Committee. The collectors moved their position every hour to avoid possible bias arising from differences in attraction and collectors’ ability at catching insects. All specimens were individually stored (dry) in tubes with silica gel. Data regarding collection time, area and capture site was recorded for each individual. Samples were transported to the Fundacion Instituto de Inmunología de Colombia’s (FIDIC) Molecular Biology laboratory for taxonomic identification and molecular analysis.

### Taxonomic and molecular identification of the mosquito species

González and Carrejo’s dichotomous keys were used for the taxonomic identification of individuals [23]; the female mosquito keys take wing and hindquarter spot patterns into account for identifying a species [23,24]. Individuals were selected for molecular confirmation by DNA barcode analysis (genetic bar code) [25]. DNA barcoding involved amplifying a 710 bp region with specific primers (LCO1490 and HCO2198) targeting a fragment of the mitochondrial cytochrome c oxidase subunit 1 gene (*COI*) [25,26]. This gene is accepted as barcode standard due to its robustness, accuracy and resolution power [27]. Sample size calculation for sequencing was based on the most abundant species, assuming a proportion of 0.5, a precision of 0.05 and a confidence interval of 95%; a minimum sample size of 163 was thus obtained. This calculation was carried out using the sampsi command in Stata (v.12). Additionally, a sub-sampling proportional to the original number of mosquitoes collected in each locality was selected for sequencing. For the samples of the less abundant species, all the individuals were selected for sequencing [28,29].

A Genomic DNA Mini Kit (Invitrogen) was used for recovering and extracting DNA from mosquito legs, following the manufacturer’s instructions. The samples were eluted in a 50 μL final volume of buffer containing 10 mM Tris-HCl and 0.1 mM EDTA at pH 9.0 and stored at -20°C until use. A ReadyMix PCR kit was used for the PCR assays in a 50 μL volume mixture containing Kapa HiFi HotStart (Kapabiosystems) and 0.2 μM of each primer. Thermal cycling conditions consisted of initial denaturing at 94°C for 1 minute, 40 amplification cycles at 94°C for 40 seconds, 52°C for 40 seconds and 72°C for 1 minute, followed by a final elongation step at 72°C for 5 minutes.

Amplification products were observed on SYBR-Safe-stained 1.5% agarose gels (Invitrogen) and visualized on a MiniBIS Pro gel doc imaging system (DNR Bio-imaging Systems). A Wizard genomic DNA purification kit was used for purification, following the supplier’s recommendations. These products were sequenced in both directions on an ABI-3730 XL sequencer (Macrogen, Seoul, South Korea).

### Evolutionary analysis of the *COI* gene

CLC Genomics Workbench (v3) (CLC Bio, Cambridge, MA, USA) was used for analyzing and assembling the electropherograms for each sample obtained by sequencing the *COI* gene. The sequences were then compared and analyzed against reference sequences available in the GenBank database (KP193458.1, JF923693.1, JF923694.1, JF923695.1, MF381713.1, MF381596.1, MF381733.1, MF381650.1, MF381626.1, MF381726.1, MF381725.1, MF381671.1, MF381589.1, MF381728.1, MF381675.1, MF381608.1, HM022406.1, KU892052.1, KU892055.1, KU892053.1, KU892051.1, KU892054.1, KU892056.1, KU892057.1, KU892058.1, KU892059.1, KU892060.1, KC555065.1, GQ918272.1, GQ918273.1, DQ076235.1, DQ076236.1). The MUSCLE method was used for aligning all the sequences [30]. The sequences obtained here were deposited in the Genbank database (accession numbers MH924354-MH924552).

DnaSP software (v5) [31] was then used for calculating the amount of segregating sites (S), the amount of segregating sites (S), singleton sites (Ss), parsimonious sites (P), haplotypes (H) and nucleotide diversity (π) per site, using all the available sequences as data set, as well as just defined populations’ sequences (i.e. Tp, Tp2 and DO). The Median-joining network algorithm (NETWORK v.5.1) [27], with the star contraction option [32], was used for evaluating the mutational pathways giving rise to the haplotypes found in the *COI* fragment, their distribution and frequencies.

### *Plasmodium* molecular detection and species identification

The mosquitoes’ heads and thoraxes were recovered to detect parasite infection in the individuals collected from the communities, thereby ensuring only having infective females and a retrieval of all of the salivary glands. They were then grouped into pools of no more than 5 individuals, giving 228 samples. DNA was then extracted, according to the previously described protocol.

The nested PCR technique was used for detecting parasites. A first amplification involved using specific primers for the 18S ribosomal RNA subunit (ssRNA); this was followed by using the first PCR product as amplification target for identifying three *Plasmodium* species (*P vivax*, *P*. *malariae* and *P*. *falciparum*), using the specific primers for each species [33]. All mixture and amplification conditions have been described previously [8].

Laboratory-bred *Anopheles albimanus* females (i.e. free of parasitic infection) were used as negative control and females from the same species infected with *Plasmodium falciparum* NF54 strain gametocytes as positive control, according to Loker & Taylor-Robinson’s artificial membrane infection method (2014) [34].

### Statistical analysis

Quantitative variables (the number of individuals) are reported, along with their respective means and standard deviations (SD). The categorical variables (time, site and ecotope) are expressed in terms of frequency and percentages, along with their 95% confidence intervals (95%CI). The χ2 test or Fisher’s exact test were used for evaluating differences between percentages, depending on the value being observed.

Biting activity was calculated as the geometric mean of the mosquitoes found per hour over the 6-hour sampling period in each locality. Statistical analysis involved using analysis of variance (ANOVA) and Bonferroni post-test adjustment for evaluating differences regarding the amount of mosquitoes collected per hour, locality and site (intradomiciliary, peridomiciliary or extradomiciliary); *p*<0.05 value were considered statistically significant. STATA software (version 12) was used for analyzing all data [35]

## Results

### Morphological identification of the mosquito species and DNA barcode analysis

During the study period we collected a total of 1,086 specimens in both areas of study; 21 of these (2.3%) lost body parts during capture and could not be morphologically classified; 1,065 specimens were taxonomically classified (using González and Carrejo’s dichotomous keys [23]). This classification highlighted *An*. *darlingi* (n = 1,057; 99.2%) as the predominant species in the population, other anopheline mosquito species collected at the study sites included *An*. *mattogrossensis* (n = 4; 0.4%). The only genus of the subfamily Culicinae found in this study was *Culex* sp. (n = 4; 0.4%) with an equal proportion of *An*. *mattgrossensis*.

Molecular identification involved selecting 214 specimens for taxonomic classification by DNA barcode; four of them morphologically classified as *Culex* sp. were identified as *Culex nigripalpus* (n = 2) and *Culex ribeirensis* (n = 2). Molecular results for the 210 remaining samples gave *An*. *darlingi*; 14 were morphologically indeterminate species, four were confirmed as *An*. *mattogrossensis* and 185 agreed with findings using the dichotomous key.

### Variation regarding mosquito abundance according to study area

**Fig 2:**
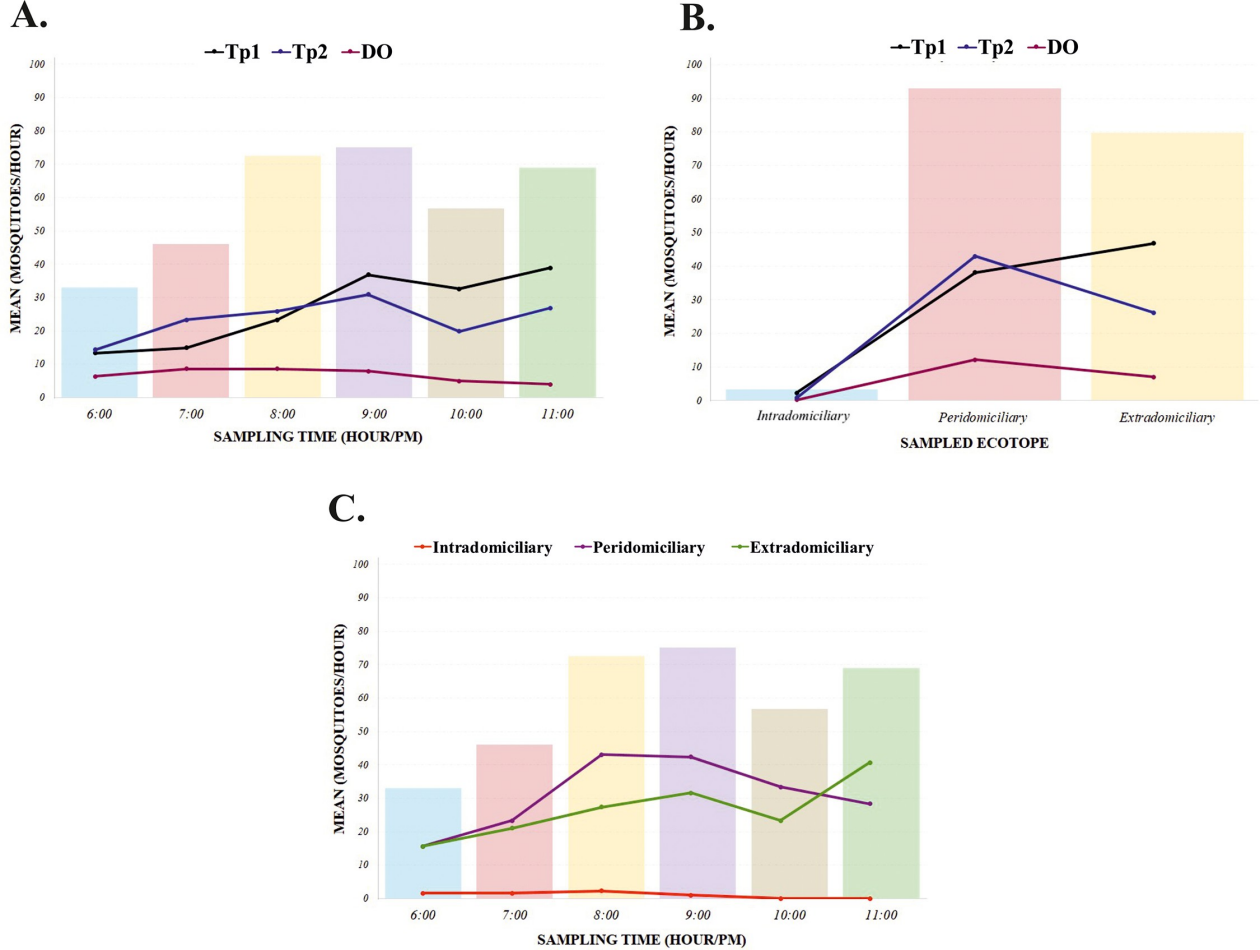
Mosquito abundance according to community, sampling time and ecotope. **A)** The mean number of mosquitoes according to sampling time and area sampled; the bars show the mean number of mosquitoes according to sampling time and continuous lines show the mean for mosquitoes per community evaluated (Tp1: Tipisca first sampling; Tp2: Tipisca second sampling; DO: Doce de Octubre). **B)** The mean number of mosquitoes according to ecotope and community; the bars show the mean for the number of mosquitoes according to ecotope and continuous lines show the mean for mosquitoes per ecotope and community evaluated (Tp1, Tp2 and DO). **C)** The mean number of mosquitoes according to ecotope and sampling time; the bars show the mean number of mosquitoes according to sampling time and continuous lines show the mean of mosquitoes per ecotope.

The study involved sampling two areas where two indigenous communities were living in the Colombian Amazon region; the abundance in Tipisca (Tp1) was of one *Culex ribeirensis* specimen (0.4%: 0.004–1.0 95%CI), one *An*. *mattogrossensis* specimen (0.4%: 0.004–1.0 95%CI) and 522 *An*. *darlingi* specimens (99.6%: 98.6–99.9 95%CI). In this area, the greatest collection frequency was found at 11:00pm (n = 116, 22.1%), with the Tp1 extradomiciliary ecotope having most collected mosquitoes (n = 280; 53.6%) (Fig 2A and S1A Fig).

A second collection took place at the Tipisca location (repeating the sampling two weeks later in the house selected for the first capture—Tp2); this sampling led to the capture of one *Culex* specimen (0.4%: 0.004–1.0 95%CI) and 419 from the *An*. *darlingi* species (99.8%: 98.6–99.9 95%CI), 9:00 pm being the hour of greatest abundance (n = 92; 21.9%), peridomiciliary being the ecotope having the highest number of mosquitoes per capture (n = 257; 61.3%) (Fig 2 and S1B Fig).

At the Doce de Octubre location we collected the smallest proportion of mosquitos in this study, in which 121 were found, two of which were classified as *Culex nigripalpus* (1.6%: 0.2–5.8 95%CI), three (2.5%: 0.5–7.0 95%CI) as *An*. *mattogrossensis* and 116 (95.9%: 90.6–98.6 95%CI) as *An*. *darlingi* (Fig 2A). The greatest abundance for this species occurred at 7:00 pm (n = 25; 20.6%) and 8:00 pm (n = 25; 20.6%). Peridomiciliary was the ecotope where the greatest capture took place (n = 73; 62.9%) (Fig 2 and S1C Fig).

### Abundance and bite rate of *An*. *darlingi* in sampling areas

Considering that *An*. *darlingi* was the most frequently found vector in the communities sampled, we compared the number of individuals of this species captured in each area, time, and ecotope. Tp1 had the highest mean for mosquitoes (mean = 26.3; SD: 14.6) regarding the areas evaluated here, followed by Tp2 (mean = 23.2; SD: 10.2) and DO (mean = 6.4; SD: 6.3). The difference in vector abundance by sampled area was evaluated using ANOVA, which showed a statistically significant result (p = 0.001). Using a Bonferroni correction, post-hoc testing showed significant differences between Tp1 and DO (*p* = 0.001) and Tp2 and DO (*p* = 0.001).

Similarly, we evaluated the times in which *An*. *darlingi* capture was most frequent in each of the sampling areas. This analysis showed that the times of highest mosquito presence in each area were 11:00 pm in Tp1 (mean = 38.6, SD: 8.3) and 9:00 pm in Tp2 (mean = 30.6; SD:12.4). Two times of highest sampling were found in DO at 7:00 pm (mean = 8.3; SD: 11.0) and 8:00 pm (mean = 8.3; SD: 10.4) (Fig 2A). The differences in means were statistically significant (*p* = 0,001; ANOVA test), and post-hoc testing using the Bonferroni correction showed significant differences in abundance between three sampling times: 9:00 pm in Tp1 and DO (p = 0.013) and Tp2 and DO (p = 0.038), 10:00 pm in Tp1 and DO (p = 0.010), and 11:00pm in Tp1 and DO (p = 0.010).

Mosquito density and biting rate was established according to the ecotope being sampled; the peridomiciliary area had the highest mean number of mosquitoes (mean = 31.0; SD: 19.2), followed by the extradomiciliary (mean = 26.6; SD: 20.6) and intradomiciliary ecotopes (mean = 1.1; SD: 1.6) (Fig 2B). Evaluating mean ecotope behavior for each community sampled showed that the extradomiciliary ecotope in Tp1 had the greatest density (mean = 46.6; SD: 19.1), followed by the peridomiciliary ecotope in Tp2 (mean = 42.8; SD: 12.7). Mean ANOVA for mosquitoes, according to ecotope and community sampled, indicated that this was significant for the peridomiciliary (*p* = 0.0041) and extradomiciliary ecotopes (*p* = 0.004) (Fig 2C).

Analyzing the time having the greatest abundance for each ecotope showed that 8:00 pm, was the time having the greatest peridomiciliary density (mean = 42.8; SD: 20.6), while this was 11:00 pm for the extradomiciliary ecotope (mean = 40.6; SD: 36.6) (Fig 2C); nevertheless, ANOVA revealed no statistically significant differences.

### Detecting *Plasmodium* species and natural infection

PCR was used for establishing three *Plasmodium* species’ infection prevalence in 224 pools of *An*. *darlingi*; 106 pools came from Tp1, 90 from Tp2 and 28 from DO. Parasite DNA was detected in 91 of 224 pools analyzed (40.6%: 34.1–47.3 95%CI); *P*. *vivax* was the mostly frequently occurring species (n = 49, 21.9%), followed by *P*. *malariae* (n = 47, 21.0%) and *P*. *falciparum* (n = 23, 10.3%). The distribution was statistically significant (*p* = 0.001).

**Fig 3:**
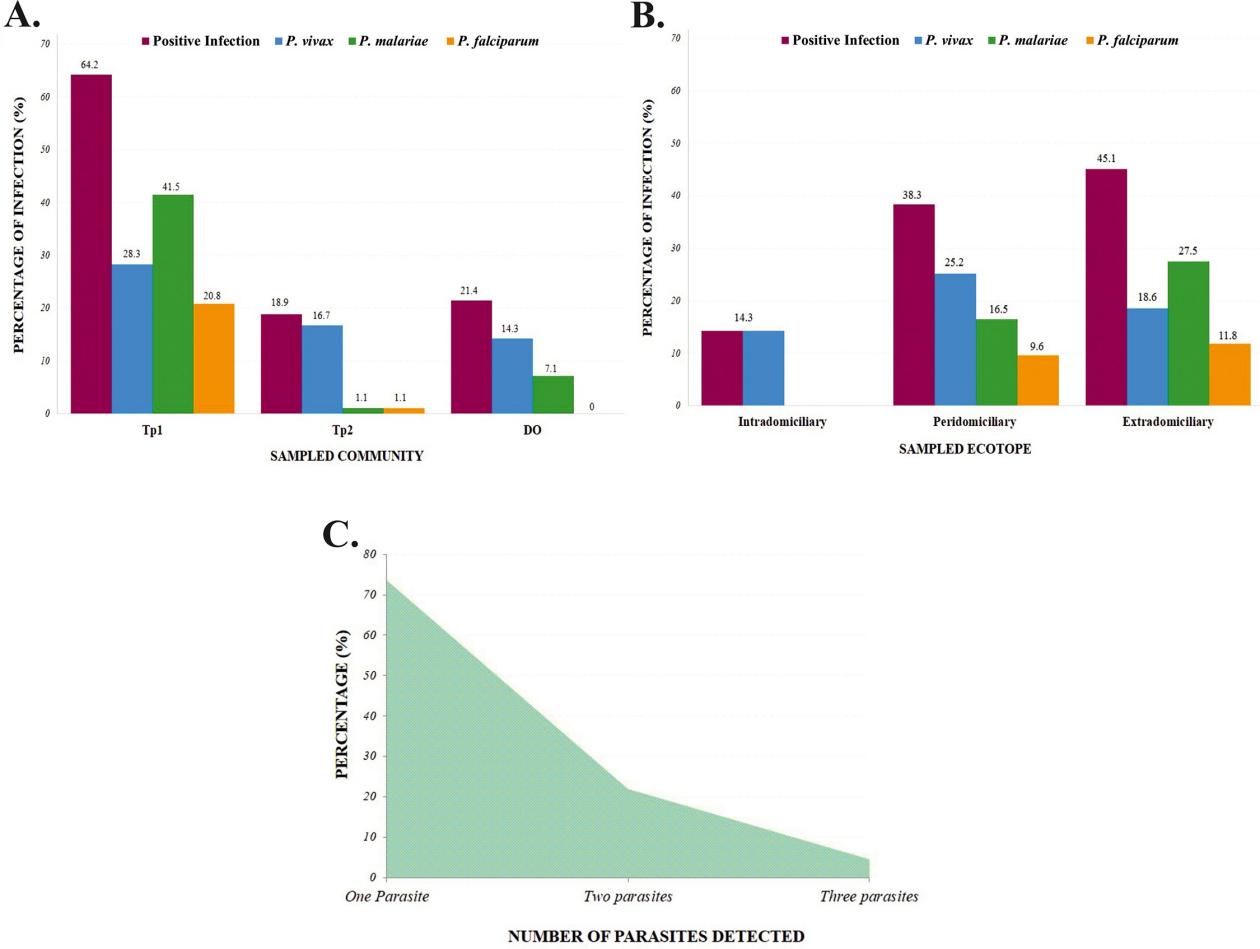
*Plasmodium* spp. infection relative frequency in the mosquito pools analyzed. **A)** Relative infection frequency for *Plasmodium* and the three *Plasmodium* species detected (*P*. *vivax*, *P*. *malariae* and *P*. *falciparum*) according to the community being sampled (Tp1, Tp2, DO). **B)** Relative frequency regarding *Plasmodium* infection and *P*. *vivax*, *P*. *malariae* and *P*. *falciparum* according to ecotope. **C)** The frequency of appearance of pools infected by one, two or three parasite species.

Tp1 was the area having the highest *Plasmodium* detection (n = 68, 64.2%) when evaluating parasite infection according to community sampled, while Tp2 had the lowest parasite prevalence (n = 17, 18.9%) (Fig 3A). Regarding parasitic species detection, *P*. *malariae* had the highest prevalence in Tp1 (n = 44; 41.5%), this being statistically significant (*p* = 0.001), whereas *P*. *vivax* had the highest frequency in Tp2 (16.7%) and DO (14.3%) (Fig 3A).

Seven of the pools analyzed came from the intradomiciliary area, this ecotope having the lowest parasite detection (n = 1, 14.3%), i.e. exclusively *P*. *vivax* (Fig 3B); 115 pools were analyzed in the peridomiciliary area, 44 of them (38.3%) having parasitic DNA and *P*. *vivax* (n = 29, 25.2%) being the most representative species. Regarding the extradomiciliary ecotope, 46 of the 102 pools evaluated (45.1%) had *Plasmodium* infection, *P*. *malarie* (n = 28; 27.5%) appearing most frequently (Fig 3B).

Regarding parasite species’ percentage in the pools, 24 (26.4%) of the pools were infected by more than one parasite species (Fig 3C), *P*. *vivax* and *P*. *malariae* having greater probability of appearing in the pools evaluated here (n = 15; 16.5%). Parasite DNA was detected in 2 (50%) of the 4 *An*. *mattogrossensis* specimens captured, being exclusively infected by *P*. *vivax*.

### Evolutionary analysis of the *COI* gene

**Fig 4:**
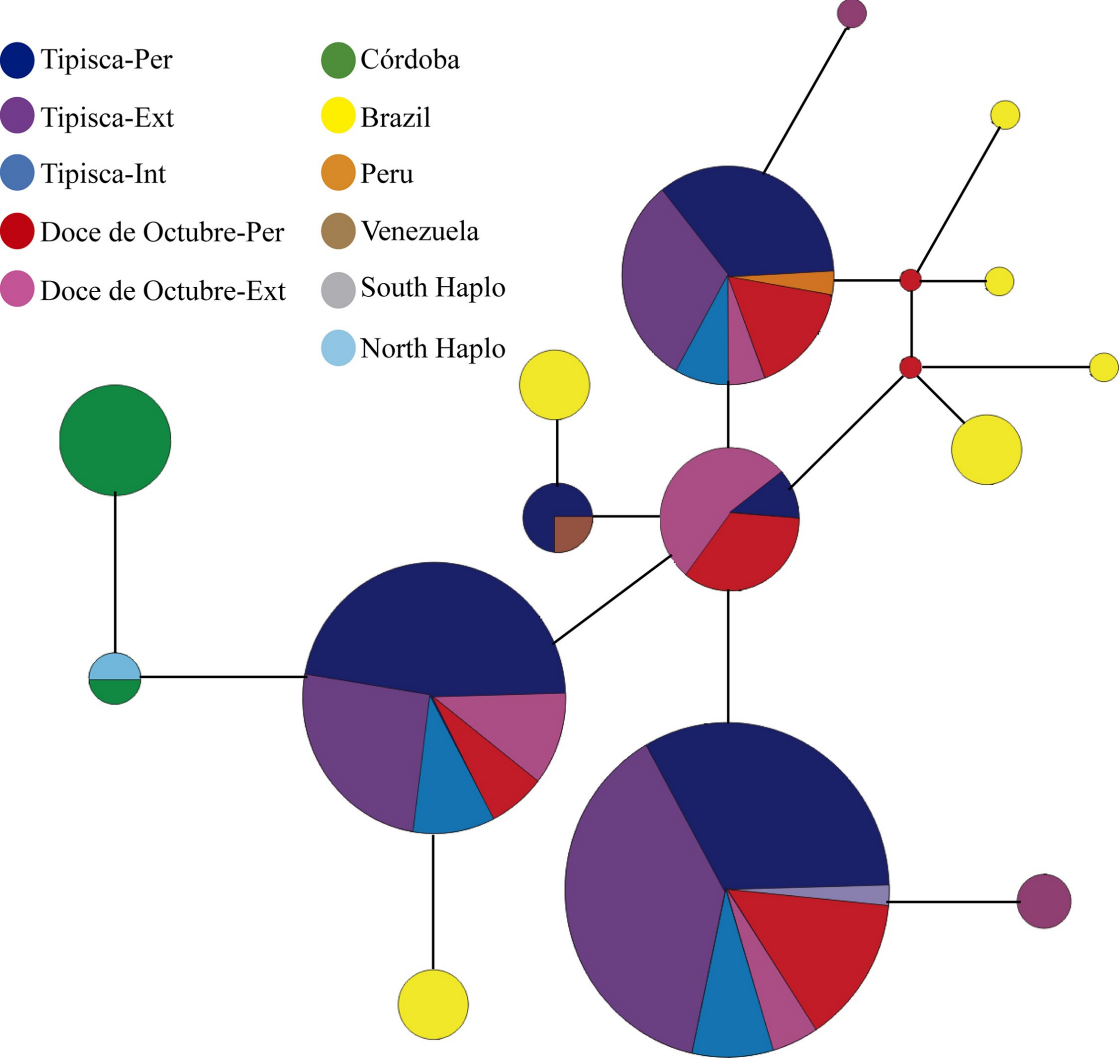
Median joining network. Fig 4 shows the *COI* haplotypes identified for the mosquitoes collected in the Colombian Amazon region; some haplotypes were included within other haplotypes, using the star contraction algorithm [32] to simplify network interpretation. Each node is a haplotype and its size indicates its frequency. The lines connect representative haplotypes, the different mutational pathways; median vectors are the ancestral sequences explaining evolutionary relationship and origin.

Analysis of the amplified *COI* gene fragment from the 199 *An*. *darlingi* sequences gave 40 single nucleotide polymorphisms (SNP) in the 596 bp fragment analyzed. Only 20 SNP were observed taking the sequences from the Colombian Amazonia region and those available in GenBank from the Córdoba department as a single data set (Colombia) whereas 26 SPNs were observed when analyzing 15 sequences from Brazil (Table 1). Regarding nucleotide diversity per site (π), the highest values were seen in the sequences from Brazil, followed by Amazonas, while sequences from the Córdoba had the lowest π value (Table 1); π values were similar regardless of sampling site. The network showed that many haplotypes were shared between sampling areas; however, some were exclusive to a particular region (Fig 4).

**Table 1:** Genetic diversity estimators.

	All	Colombia	Amazonas	Tp	Tp PE	Tp EX	Tp IN	DO	DO PE	DO EX	Córdoba	Brazil
**N**	231	210	199	145	69	61	15	52	29	23	11	15
**Site**	595	606	666	666	666	666	666	666	666	666	606	643
**Ss**	40	20	22	13	13	11	10	19	12	15	8	26
**S**	21	9	8	2	3	3	2	7	2	5	7	12
**Ps**	19	11	14	11	10	8	8	12	10	10	1	14
**H**	35	18	28	9	8	5	5	21	12	14	7	14
**Hd**	0.800	0.764	0.770	0.699	0.756	0.615	0.771	0.895	0.796	0.949	0.873	0.990
**π (SD)**	0.00365 (0.0002)	0.00297 (0.0002)	0.0055 (0.0002)	0.00548 (0.0002)	0.00599 (0.0003)	0.00474 (0.0004)	0.00578 (0.0007)	0.00537 (0.0005)	0.00490 (0.0007)	0.00574 (0.0007)	0.00264 (0.0007)	0.01059 (0.0009)

The available sequences were used for genetic diversity estimators. n: the amount of sequences analyzed, Site: the total of sites analyzed, excluding gaps. Ss: the total of segregating sites, S: the total of singleton sites, Ps: the total of parsimonious sites, H: the number of haplotypes, π: nucleotide diversity by site, SD: standard deviation, Hd: haplotype diversity. Abbreviations: Tipisca (Tp); Tipisca peridomiciliary (Tp PE); Tipisca extradomiciliary (Tp EX); Tipisca intradomiciliary (Tp IN); Doce de Octubre (DO); Doce de octubre peridomiciliary (DO PE); Doce de octubre Extradomiciliary (DO EX).

## Discussion

The diversity of mosquitoes from the *Anopheles* genus in Colombia is mainly favored by a wide variety of habitats, providing environmental conditions for mosquito development, dispersion and persistence [33]. Although some primary vectors are widely distributed throughout Colombia’s five regions, their biting patterns and ecology may vary; this means that such aspects must be studied to improve the efficiency of currently used control methods [29,36].

The Anopheline diversity in the areas sampled in this study was lower than that reported from other sites in the Colombian Amazon region where seven species from the subgenus *Nyssorhynchus* have been found (*An*. *oswaldoi*, *An*. *nuneztovary*, *An*. *triannulatus*, *An*. *rangeli*, *An*. *evansae*, *An*. *benarrochi* and *An*. *dunhami*) along with six from the subgenus *Anopheles* (*An*. *neomaculipalpus*, *An*. *punctimacula*, *An*. *mediopunctatus*, *An*. *mattogrossensis*, *An*. *apimacula* and *An*. *peryassui*) [14,15,37]. However, these vectors’ sparse diversity should be compared to other research carried out at different times in the year to ascertain whether the species change and to determine the environmental or climatic factors directly affecting this at each of the ecotopes sampled in this project.

This study showed that *An*. *darlingi* was the dominant species at the three sampling sites; it is considered one of the most effective primary vectors in the Amazon region due to its recognized anthropophilic host tendency, great abundance in certain areas, being susceptible to infection by several *Plasmodium* species and sometimes being endophagic [38]. It can adapt to several types of habitat, including anthropic activity-related hatcheries [39–41]. The dominance of certain species of anophelines has been associated with environmental transformations, especially in the Brazilian Amazon region where changes in these ecosystems have been reported to have a direct impact on the presence of other mosquito species from this genus, some species even beginning to disappear because of greater exposure to light or a lack of aquatic plants in the breeding grounds frequented by these mosquitoes, such conditions affecting their immature forms’ development [38,42].

The amount of mosquitoes gradually decreases when such transition periods are over, mainly due to a loss of breeding areas caused by the imminent arrival of drought [43]. The latter was observed during the second sampling in Tp2 for *An*. *darlingi*, directly affecting the number of females. Nonetheless, and despite sampling having taken place only in the afternoon, the number of mosquitoes captured is overall higher than that reported in other studies in which the implementation of the same method has led to similar numbers after several months or years [44–46]. This indicates that in these zones of the Colombian Amazon, *An*. *darlingi* can rapidly reach high densities, which makes it necessary to implement mosquito population control measures at times in which density is lower. In general, this low density is reached during drought periods, and control measures implemented at that time would limit population abundance after rains [47].

*An*. *darlingi* exhibited an exophilic and exophagic behavior pattern in the study areas because of its increased presence in the peri- and extra-domiciliary areas. Biting activity was constant during the night, peaking after 21:00 p.m. Such marked presence in the areas surrounding human settlements has been discussed previously and must be determined by ascertaining different variables, such as insecticide use within houses (administered by the area’s control strategies), using long-lasting, insecticide-treated bed nets (LLIN), the type of construction and the area where sampling was carried out [48–50]. On the other hand, our results could indicate possible changes in *An*. *darlingi*’s behavior. Previous reports from this sampling area had shown that this mosquito behaved as an endophage, increasing its biting activity late at night. This area is inhabited by the Huitoto and Ticuna ethnic groups, which obtain sustenance from fishing and agriculture and are generally not active after 9:00 pm, instead resting under mosquito nets in their homes [14]. The exophagy of *An*. *darlingi* suggested by our results does not appear to be determined by the customs of the inhabitants in this part of the Amazon. Nonetheless, the results obtained here require support from future studies that should include information regarding the population’s behavior, thereby allowing for an appropriate determination of the relationship between mosquito behavior and human occupation.

The *COI* marker for analyzing diversity revealed no differences between sampling areas and their ecotypes, thereby suggesting considerable genetic exchange between *An*. *darlingi* mosquitoes from both localities, perhaps caused by similar environmental and geographic conditions in Tipisca and Doce de Octubre. Previous studies have highlighted greater micro-geographic genetic diversity by habitat type and season, some related to recent forest degradation in areas near rural townships in Brazil and Perú having abrupt environmental conditions changing/affecting mosquito reproduction rate, fitness and survival [51,52].

Although *An*. *darlingi* abundance differed in each community, no marked differences were found regarding genetic diversity (evaluated via *COI* gene) so our population’s results would suggest that such differences did not seem to be determined by variations in *An*. *darlingi* population structure. Previous studies have observed that variations between populations or communities regarding the amount of mosquitoes could be partly explained by behavioral plasticity in response to environmental variations at sampling sites [53].

It was observed that the sequences obtained in the Colombian Amazon region were related to those from the Brazilian Amazon when comparing our population structure results regarding the *COI* marker with reports from Central and South America (Table 1). Such *An*. *darlingi* population discrepancies were related to isolation due to the distance between populations and to geological events which have isolated mosquitoes from Central America from the rest of the South American Anophelines [54].

However, the similarity between the Brazilian haplotypes and those in this study may have been due to demographic or ecological events avoiding these mosquitoes’ loss of population identity [55]. Our results agreed with previously documented data; a genetic difference has arisen between *An*. *darlingi* populations from Colombia’s north-western and south-eastern regions, those from Colombia’s eastern plains and the Colombian Amazonas region being genetically closer to populations from other South American countries [56].

The three *Plasmodium* species reported for Colombia were found in *An*. *darlingi* females in the study areas, as reported for communities living in northern Brazil, southern Venezuela and French Guyana where *P*. *malariae* prevalence in the females from these populations is lower than reported for *P*. *vivax* and *P*. *falciparum* [57–59]. Our results highlighted the high percentage of *P*. *malariae*-infected *An*. *darlingi* females, although a similar percentage of *P*. *malariae*- and *P*. *vivax*-infected individuals was observed in the study areas, thereby agreeing with previous epidemiological studies in these areas (Fig 3) [8]. Previous studies have given a possible explanation for the low probability of finding *P*. *malariae* in mosquitoes due to their prolonged sporogonic cycle in the females’ mid-intestine which can last for up to three weeks, thereby decreasing the probability of infective females being produced [60]. This biological aspect of *P*. *malariae* may induces false negatives in CSP-ELISA since this immunoassay depends directly on the amount of antigen in a sample [61].

Molecular techniques such as PCR help determine the presence of any *Plasmodium sp*. species in Anophelines with greater precision since small amounts of parasite can be detected, thus helping to monitor and evaluate malarial transmission [62]. The high prevalence of *Plasmodium* sp. infection in *An*. *darlingi* in such remote areas where humans are present suggests that these anthropophilic mosquitoes have a high vectorial capability and cause asymptomatic parasitemia while maintaining the malarial life/cycle [63].

No robust evidence has been found for categorizing high *P*. *malariae* prevalence as a zoonosis in the study areas; however, it is worth mentioning the high frequency of infected females being collected around the houses’ outer areas (extradomiciliary and peridomiciliary) where New World primates circulate, given the dwellings’ proximity to Amazon rainforest flora. There is evidence of females belonging to the *P*. *malariae* -infected *An*. *fluminensis*, *An*. *pseudomaculilapus* and *An*. *maculipes* species [64] in areas of the Brazilian Atlantic forest where the inhabitants work near this forest or make sporadic incursions, leading to the speculation that this disease behaves like a jungle zoonosis given the entomological scenario and social data collected to date [64,65].

On the other hand, *An*. *mattogrossensis* is usually collected in the Amazon region in countries like Perú, Brazil and Colombia [39,66,67]. Its poor abundance and zoophilic behavior mean that it is not considered an important species for malarial transmission, although it can be found infected by *P*. *falciparum* and *P*. *vivax* in some Brazilian localities. This is the first report concerning Colombia of *An*. *mattogrossensis* infected by a malaria-producing parasite (*P*. *vivax*) in humans. It has been categorized as a potential reservoir for this parasite species; such finding agrees with other work since its abundance was lower than that for other Anopheline species found [66,68,69]. These results suggest that monitoring studies must be designed which are mainly aimed at identifying mosquitoes such as *An*. *mattogrossensis*, thereby enabling suitable characterization of the other species involved in malarial transmission in the Colombian amazon region [67].

The present research highlights the need for investigating the mosquito species in the Colombian Amazon area throughout the year, observing their replacement and prevalence as well as making a correct association with aspects of their biology, biting activity and *Plasmodium* parasite infectivity. In short, the composition of Anopheline species in the study areas has been described, as has *An*. *darlingi*’s important role in malarial transmission since it was the most dominant and abundant species, having the capability of transmitting three different etiological causative agents of malaria.

Due to this vector’s preference for peridomiciliary and extradomiciliary areas, it would be interesting to establish whether such behavior is mediated by some vector control mechanism, environmental change or if the sampled communities’ social aspects are involved. The areas’ inhabitants work-related practices near these sites thus become risk factors according to the entomological data; some approaches for controlling this vector must be adopted which include using LLINs, personal repellents, insecticides for use on livestock or the search for potential hatcheries using larvicides for decreasing *An*. *darlingi* abundance [53].

## Supporting information

S1 Fig A description of the number of mosquitoes captured per community, sampling time and ecotope; the bars show the number of mosquitoes and continuous lines show the amount of mosquitoes captured per ecotope. **A)** Tp1 **B)** Tp2 and **C)** DO communities.
(TIF)
