# Supplementary figures and images for: Behavior and abundance of Anopheles darlingi in communities living in the Colombian Amazon riverside
Source: PLoS One. 2019 Mar 7;14(3):e0213335. doi: 10.1371/journal.pone.0213335 (PMC6405047; doi:10.1371/journal.pone.0213335)

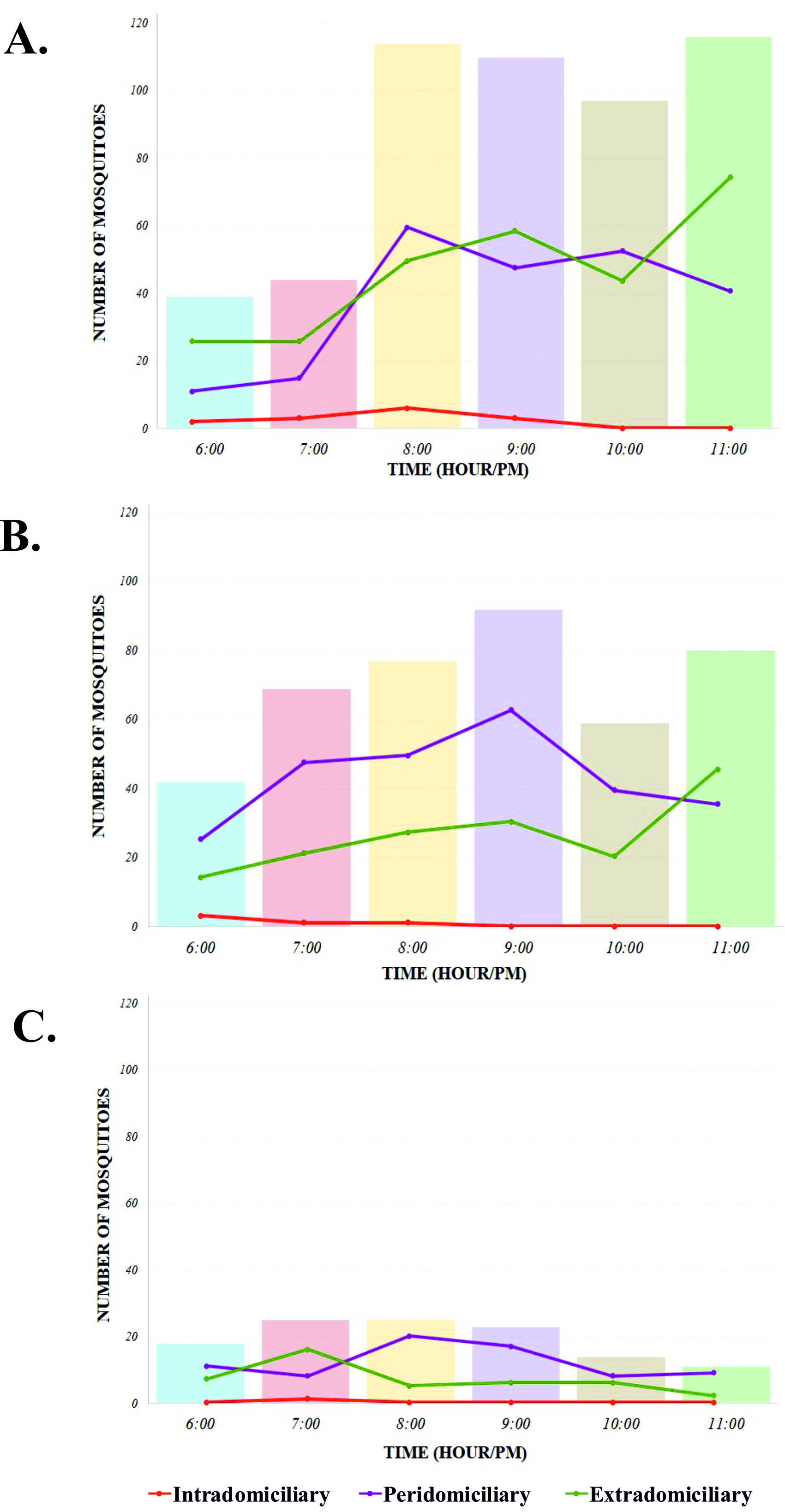

Supplement: S1 Fig — A) Tp1 B) Tp2 and C) DO communities. (TIF) [file pone.0213335.s001.tif]
